# Draft Genome Sequence of the Astaxanthin-Producing Microalga Haematococcus lacustris Strain NIES-144

**DOI:** 10.1128/MRA.00128-20

**Published:** 2020-06-04

**Authors:** Daichi Morimoto, Takashi Yoshida, Shigeki Sawayama

**Affiliations:** a Graduate School of Agriculture, Kyoto University, Kitashirakawa Oiwake-cho, Sakyo-ku, Kyoto, Japan; Vanderbilt University

## Abstract

Haematococcus lacustris is an industrially important eukaryotic microalga that is thought to be a great source of natural astaxanthin with strong antioxidant activity. Here, we report the draft assembly and annotation results of the genome of *H. lacustris* NIES-144. These data will expand our knowledge of the molecular biological features of this microalga.

## ANNOUNCEMENT

*Haematococcus* is a genus of eukaryotic *Chlorophyceae* microalgae that can form a red immotile cyst and accumulate the highest content of natural astaxanthin reported to date under stress conditions (1). Although these microalgae have been studied as a natural resource for astaxanthin, which is a high-value carotenoid with strong antioxidant activity (2), the genomic information is limited to H. lacustris strain SAG192.80 (3).

To expand our molecular biological knowledge of these industrially important microalgae, we determined the draft genome sequence of *H. lacustris* NIES-144, which was obtained from the Natural Institute for Environmental Studies (NIES, Japan). *H. lacustris* NIES-144 was cultured in C medium (4) under 14/10-h light/dark photocycles at 25°C. Extraction of genomic DNA from *Haematococcus* cells was performed using a FastDNA Spin kit for soil (MP Biomedical, USA). Paired-end and mate pair libraries (3 kb and 10 kb, respectively) were prepared using a combination of the Covaris (USA) sonicator and the TruSeq DNA LT sample prep kit or the Nextera mate pair sample preparation kit (Illumina), respectively. The paired-end library was sequenced using the TruSeq rapid sequencing by synthesis (SBS) kit on the Illumina HiSeq 2500, while the mate pair library was sequenced using the TruSeq SBS kit v3 on the Illumina HiSeq 2000 platform.

The mate pair reads (average, 154,807,864 reads) were processed with cutadapt 1.2.1 (5) to remove adapter sequences. The paired-end reads (215,289,986 reads) and trimmed mate pair reads (average, 105,701,143 reads) were assembled into 9,693 scaffolds with a total length of 172 Mb (genome coverage, 186×; GC content, 58.4%; *N*_50_ scaffold length, 38,941 bp) using ALLPATHS-LG R45226 (6) with the following parameters: GENOME_SIZE: 125,000,000; FRAG_COVERAGE: 100; JUMP_COVERAGE: 100; and HAPLOIDFY: True. The completeness of the draft genome was 57.7% based on the Benchmarking Universal Single-Copy Orthologs (BUSCO) software v3.1.0 (eukaryota_odb9 database) (7). Prior to gene structure prediction, the repeat sequences of the *H. lacustris* NIES-144 genome were identified and masked by RepeatMasker v4.0.9 (8) with default parameters. The gene structure of the masked *Haematococcus* genome was predicted by using MAKER v2.31.10 (9) in collaboration with AUGUSTUS 3.3.2 (10), SNAP v2006-07-28 (11), and GeneMark-ES 4.3.0 (12) (model parameters, *Chlamydomonas*, Arabidopsis thaliana, and Chlamydomonas reinhardtii, respectively). For RNA and protein homology evidence in the MAKER prediction, we also recruited the transcriptome data of *H. lacustris* NIES-144 (SRA accession number SRX3729494) (13) and the protein sequences of representative eukaryotic species, including *H. lacustris* strain SAG192.80 (3). A total of 13,309 genes were functionally annotated for *H. lacustris* NIES-144 by BLASTp analysis against the UniProtKB SWISS-PROT and TrEMBL databases (14) with E value thresholds of <1.0 × 10^−5^ and InterProScan v5.36-75.0 (15) analysis against the Pfam database (16). Also, 277 tRNAs were predicted using tRNAscan-SE v2.0 (17). This genome will provide the prerequisite information for genetic engineering and spur the further development of efficient astaxanthin production by this microalga.

### Data availability.

This whole-genome shotgun project has been deposited at DDBJ/ENA/GenBank under BioProject number PRJDB8952 (BioSample number SAMD00192397). This version of the project has the accession number BLLF00000000 and consists of sequences deposited under the accession numbers BLLF01000001 to BLLF01009693. The raw reads can be accessed under the SRA accession number DRP005830.

## References

[1] Liu J, Sun Z, Gerken H, Liu Z, Jiang Y, Chen F 2014 *Chlorella zofingiensis* as an alternative microalgal producer of astaxanthin: biology and industrial potential. Mar Drugs 12:3487–3515. doi:10.3390/md12063487. 24918452 PMC4071588

[2] Ambati RR, Phang S-M, Ravi S, Aswathanarayana RG 2014 Astaxanthin: sources, extraction, stability, biological activities and its commercial applications—a review. Mar Drugs 12:128–152. doi:10.3390/md12010128. 24402174 PMC3917265

[3] Luo Q, Bian C, Tao M, Huang Y, Zheng Y, Lv Y, Li J, Wang C, You X, Jia B, Xu J, Li J, Li Z, Shi Q, Hu Z 2019 Genome and transcriptome sequencing of the astaxanthin-producing green microalga, *Haematococcus pluvialis*. Genome Biol Evol 11:166–173. doi:10.1093/gbe/evy263. 30496415 PMC6330051

[4] Ichimura T 1971 Sexual cell division and conjugation-papilla formation in sexual reproduction of *Closterium strigosum*. Proc 7th Int Symp Seaweed Res 1971:208–214.

[5] Martin M 2011 Cutadapt removes adapter sequences from high-throughput sequencing reads. EMBnet J 17:10–12. doi:10.14806/ej.17.1.200.

[6] Gnerre S, MacCallum I, Przybylski D, Ribeiro FJ, Burton JN, Walker BJ, Sharpe T, Hall G, Shea TP, Sykes S, Berlin AM, Aird D, Costello M, Daza R, Williams L, Nicol R, Gnirke A, Nusbaum C, Lander ES, Jaffe DB 2011 High-quality draft assemblies of mammalian genomes from massively parallel sequence data. Proc Natl Acad Sci U S A 108:1513–1518. doi:10.1073/pnas.1017351108. 21187386 PMC3029755

[7] Simão FA, Waterhouse RM, Ioannidis P, Kriventseva EV, Zdobnov EM 2015 BUSCO: assessing genome assembly and annotation completeness with single-copy orthologs. Bioinformatics 31:3210–3212. doi:10.1093/bioinformatics/btv351. 26059717

[8] Tarailo-Graovac M, Chen N 2009 Using RepeatMasker to identify repetitive elements in genomic sequences. Curr Protoc Bioinformatics 25:4.10.1–4.10.14. doi:10.1002/0471250953.bi0410s25. 19274634

[9] Cantarel BL, Korf I, Robb SMC, Parra G, Ross E, Moore B, Holt C, Sánchez Alvarado A, Yandell M 2008 MAKER: an easy-to-use annotation pipeline designed for emerging model organism genomes. Genome Res 18:188–196. doi:10.1101/gr.6743907. 18025269 PMC2134774

[10] Stanke M, Schöffmann O, Morgenstern B, Waack S 2006 Gene prediction in eukaryotes with a generalized hidden Markov model that uses hints from external sources. BMC Bioinformatics 7:62. doi:10.1186/1471-2105-7-62. 16469098 PMC1409804

[11] Johnson AD, Handsaker RE, Pulit SL, Nizzari MM, O'Donnell CJ, de Bakker PIW 2008 SNAP: a Web-based tool for identification and annotation of proxy SNPs using HapMap. Bioinformatics 24:2938–2939. doi:10.1093/bioinformatics/btn564. 18974171 PMC2720775

[12] Ter-Hovhannisyan V, Lomsadze A, Chernoff YO, Borodovsky M 2008 Gene prediction in novel fungal genomes using an ab initio algorithm with unsupervised training. Genome Res 18:1979–1990. doi:10.1101/gr.081612.108. 18757608 PMC2593577

[13] Lee C, Ahn J-W, Kim J-B, Kim JY, Choi Y-E 2018 Comparative transcriptome analysis of *Haematococcus pluvialis* on astaxanthin biosynthesis in response to irradiation with red or blue LED wavelength. World J Microbiol Biotechnol 34:96. doi:10.1007/s11274-018-2459-y. 29916185

[14] Boeckmann B, Bairoch A, Apweiler R, Blatter M-C, Estreicher A, Gasteiger E, Martin MJ, Michoud K, O'Donovan C, Phan I, Pilbout S, Schneider M 2003 The SWISS-PROT protein knowledgebase and its supplement TrEMBL in 2003. Nucleic Acids Res 31:365–370. doi:10.1093/nar/gkg095. 12520024 PMC165542

[15] Jones P, Binns D, Chang H-Y, Fraser M, Li W, McAnulla C, McWilliam H, Maslen J, Mitchell A, Nuka G, Pesseat S, Quinn AF, Sangrador-Vegas A, Scheremetjew M, Yong S-Y, Lopez R, Hunter S 2014 InterProScan 5: genome-scale protein function classification. Bioinformatics 30:1236–1240. doi:10.1093/bioinformatics/btu031. 24451626 PMC3998142

[16] El-Gebali S, Mistry J, Bateman A, Eddy SR, Luciani A, Potter SC, Qureshi M, Richardson LJ, Salazar GA, Smart A, Sonnhammer ELL, Hirsh L, Paladin L, Piovesan D, Tosatto SCE, Finn RD 2019 The Pfam protein families database in 2019. Nucleic Acids Res 47:D427–D432. doi:10.1093/nar/gky995. 30357350 PMC6324024

[17] Lowe TM, Eddy SR 1997 tRNAscan-SE: a program for improved detection of transfer RNA genes in genomic sequence. Nucleic Acids Res 25:955–964. doi:10.1093/nar/25.5.955. 9023104 PMC146525

